# Health-Related Quality of Life and Its Correlates among Chinese Migrants in Small- and Medium-Sized Enterprises in Two Cities of Guangdong

**DOI:** 10.1371/journal.pone.0083315

**Published:** 2014-01-02

**Authors:** Liming Lu, Guanyang Zou, Zhi Zeng, Lu Han, Yan Guo, Li Ling

**Affiliations:** 1 Faculty of Medical Statistics and Epidemiology, School of Public Health, Sun Yat-sen University, Guangzhou, China; 2 Sun Yat-sen Center for Migrant Health Policy, Sun Yat-sen University, Guangzhou, China; 3 Institute for International Health and Development, Queen Margaret University, Edinburgh, Scotland; The Ohio State University, United States of America

## Abstract

**Objectives:**

To explore the relationship between health-related quality of life (HRQOL) status and associated factors among rural-to-urban migrants in China.

**Methods:**

A cross-sectional survey was conducted with 856 rural-to-urban migrants working at small- and medium-size enterprises (SMEs) in Shenzhen and Zhongshan City in 2012. Andersen's behavioral model was used as a theoretical framework to exam the relationships among factors affecting HRQOL. Analysis was performed using structural equation modeling (SEM).

**Results:**

Workers with statutory working hours, higher wages and less migrant experience had higher HRQOL scores. Need (contracting a disease in the past two weeks and perception of needing health service) had the greatest total effect on HRQOL (*β* = −0.78), followed by enabling (labor contract, insurance purchase, income, physical examination during work and training) (*β* = 0.40), predisposing (age, family separation, education) (*β* = 0.22) and health practices and use of health service (physical exercise weekly, health check-up and use of protective equipments) (*β* = −0.20).

**Conclusions:**

Priority should be given to satisfy the needs of migrant workers, and improve the enabling resources.

## Introduction

China has experienced dramatic industrialization, urbanization, and economic growth over the last three decades [1]. As the important drivers of economic growth, small- and medium-sized enterprises (SMEs) employ 75 percent of China's total workforce. SMEs contribute 60 percent of its GDP, and comprise over 99.5 percent of China's businesses [2]. SMEs host most of the rural-to-urban migrants, who migrate from less developed areas to more developed areas for better livelihood. By 2011, the migrant population had reached 221 million, and is estimated to have an annual increase of 10% [3]–[5]. Access to health care and health-related quality of life (HRQOL) of migrants has become increasingly important in China [6]. Rural-to-urban migrants, particularly those employed at SMEs, are more vulnerable for occupational health risks and reduced HRQOL. SMEs often failed to provide adequate health protection for migrant workers due to insufficient funds, poor management and supervision [7], [8]. Due to the household registration system, migrants are often deprived of local public health services and medical insurance schemes [9]. Migrants often live in poor conditions and work in highly intensive environment [8]–[10].

HRQOL is a broad concept consisting of physical health and psychological well-being. Perceived HRQOL can well predict subsequent physical illness and psychological disorders [11]. Studies found that the HRQOL of rural-to-urban migrants was poorer than that of urban residents [12]–[14]. HRQOL could be influenced by multiple factors, such as demographic characteristics, personal capacity, personal social security, work protection awareness, medical care needs and personal health practices[15]–[18]. Most studies on the health of Chinese migrants have focused on particular aspects of health status (such as infectious diseases, disease control, occupational hazards) [5]–[10]. Few studies to date have been published on the integrated and comprehensive evaluation of migrant workers' HRQOL. This study aims to explore the correlation and recursive relationships between these key factors and the HRQOL outcome.

## Methodology

### Theoretical Framework

**Figure 1:**
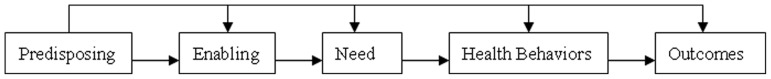
Andersen's behavioral model.

Andersen's behavioral model is employed to explore factors associated with HRQOL among rural-to-urban migrants [19]. This model provides understanding of the social, behavioral, and attitudinal determinants of health outcomes (Fig. 1). In this model, predisposing factors include demographic characteristics, social structure factors, and beliefs [20]. Enabling factors are resources at the individual, family, and community levels that may enable individuals to use health resources [20]. Needs, both perceived and evaluated, are influenced by the predisposing and enabling factors [20]. The interrelationship between these three categories will, in turn, determine health behavior (i.e., personal health practices and use of health services), and health outcomes (HRQOL) [20].

To date, few studies have examined Andersen's model by exploring how the latent variables (e.g. predisposing, enabling, need, health behavior) interrelate with health outcomes of rural-to-urban migrants in SMEs in China. The latent variables are not directly measured, but reflected by the observed variables. Based on Andersen's behavioral model, we used structural equation modeling (SEM) to build up an analysis model of HRQOL of rural-to-urban migrants and its associated factors.

### Study setting

Guandong province has the largest number of rural-to-urban migrants in China [21]. The study was conducted in Shenzhen and Zhongshan, two of the cities in the Pearl River Delta Areas of Guangdong in spring of 2012. Shenzhen had higher GDP per capita and proportion of migrant population than Zhongshan (Table 1)[22]. A medium-sized enterprise has an operating income of RMB 20∼400 million and 300∼1000 employees; a small-sized enterprise has an operating income of RMB 3∼20 million and 20∼300 employees; a miniature enterprise has less than RMB 3 million and less than 20 employees[23].

**Table 1:** General information of study cities (2011).

Cities	GDP per capita(RMB)	Population (10000 persons)	Migrant population (10000 persons)	Ratio of migrant to resident population (%)
Guangdong	50807	10432.05	3680.66	35.28
Shenzhen	110421	1035.84	851.50	82.20
Zhongshan	70014	312.13	176.15	56.43

Available from: http://www.gdstats.gov.cn/tjnj/2012/ml1.htm

GDP: gross domestic product; RMB: renminbi (China's currency in circulation, the unit of the RMB is the yuan).

### Sampling

A stratified sampling method was employed to select the study participants from miniature, small and medium sized enterprises in each city. Based on the random sampling estimation [24],
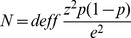
, 95% confidence(z), *P* = 0.5, design effect = 2.0, 
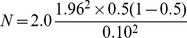
, each city required 192 samples. Considering the loss to follow rate of 20%, and satisfying multi-variable and multi-model analysis (normally 1.7 times the univariable and single-model analysis), we needed N = 192×1.7/0.8 = 408 in each city. In total, we needed at least 816 samples from 2 cities. Based on the proportion of employees in the medium, small and miniature-sized group in both cities (6∶5∶1) [25], the required sample size was 204, 170 and 34 for each group in each city.

Enterprises which provided consent to the study were randomly selected from each group of the district administration's list based on a computer-generated random number. The county CDC (or occupational health institute) assisted in the recruitment of the local enterprises

### Samples

We recruited first-line production workers 1)without a permanent residency in Shenzhen or Zhongshan; 2) having resided in the study areas for at least one month; 3) able to provide written informed consent. We excluded 1) team leaders or management personnel; 2) having difficulty in communications, such as reading or answering the study questionnaires. In total, we recruited 867 eligible migrant workers, with the response rate of 100%. We included 856 questionnaires for analysis as 11 had serious missing data or logical problems. In Shenzhen, we have selected 4 medium-sized, 9 small-sized, 5 miniature-sized enterprises, with 198, 157 and 71 migrant workers, respectively; in Zhongshan, we have selected 3 medium-sized, 4 small-sized, 3 miniature-sized enterprises, with 253, 144 and 33 migrant workers, respectively.

### Ethics Statement

The study was approved by the Institutional Review Board of the School of Public Health, Sun Yat-sen University. Written informed consent was obtained from all the study participants. Data was preserved in Sun Yat-sen Center for Migrant Health Policy (CMHP) of Sun Yat-sen University.

### Survey instrument

The 12-item Short Form Health Survey (SF-12) was used to collect information on the HRQOL of rural-to-urban migrants. SF-12 has good reliability and validity in most studies [26]–[28]. The instrument contains eight dimensions extracted from 12 items (Table 2) [28]–[30]. Two factors (Physical Component Summary (PCS) and Mental Component Summary (MCS)) can be calculated from the 8 dimensions by applying scoring algorithms with weighted item responses. The higher the score, the better the perceived HRQOL [31].

**Table 2:** SF-12 item and summary descriptive statistics (N = 856).

Item description (scale)	Mean (SD)	95% CI	Response frequencies (%)					
			1	2	3	4	5	6
Health in general (GH)*	3.62(1.09)	3.54–3.69	13.1	32.6	23.6	24.9	5.8	
Moderate activities (PF1)	2.91(0.32)	2.89–2.93	10.9	17.5	71.6			
Climb several flights (PF2)	2.93(0.28)	2.91–2.95	12.7	19.3	68.0			
Accomplished less (RP1)	1.90(0.30)	1.88–1.92	19.8	80.2				
Limited kind of work (RP2)	1.90(0.29)	1.88–1.92	19.4	80.6				
Pain interferes (BP)*	1.81(0.39)	1.78–1.84	25.2	25.4	26.6	15.5	7.3	
Accomplished less (RE1)	1.87(0.33)	1.85–1.89	19.2	80.8				
Not careful (RE2)	5.65(0.66)	5.61–5.70	13.2	86.8				
Peaceful (MH1)*	4.41(1.25)	4.32–4.49	13.9	39.7	12.2	13.7	13.2	7.3
Blue/sad (MH2)	4.38(1.19)	4.30–4.46	5.5	6.8	11.6	15.1	16.8	44.2
Energy (VT)*	4.98(0.95)	4.92–5.04	11.8	38.2	17.7	12.3	13.2	6.8
Social time (SF)	5.59(0.86)	5.53–5.65	5.5	6.8	11.6	15.1	16.8	44.2
**Summary statistics**	**PCS**	**MCS**						
Mean (SD)	53.43	51.31						
95% CI	53.07–53.79	50.79–51.83						
Cronbach's α	0.62	0.65						
Skewness	−1.15	−1.01						
Minimum (% floor)	31(0.2)	23(0.1)						
Maximum (% ceiling)	65(0.2)	66(0.1)						

Item recorded in order to make all response frequencies in the same direction. Now for all 12 items higher scores indicate better condition.

Abbreviations: GH: General Health, PF: Physical Functioning, RP: Role Physical, BP: Bodily Pain, RE: Role Emotional, MH: Mental Health, VT: Vitality, SF: Social Functioning, SD: standard deviation.

In addition to the SF-12, a structured questionnaire was developed in consultation with experts of public health, social science, public management. This questionnaire covered questions on demographic characteristics, occupational history, labor and social security, health status, and health services utilization. These indicators were chosen on the principle of ‘best fit’ to components of Andersen's behavioral model.

### Measures

To test Andersen's model, firstly, we identified measured indicators for the five proposed latent variables from the questionnaire. The first *Latent variable* is Predisposing factors, the corresponding *measured variables* of which are age, family separation, education. Based on the average age of Chinese migrants (27.9) in 2010 [7], the age variable was divided into two categories. The second *Latent variable* is Enabling resources, the corresponding *measured variables* of which are average monthly income, physical examination, length of labor contract, type of insurance and training on occupational health and safety. The third *Latent variable* is Need, the corresponding *measured variables* of which are perceived and evaluated need. Perceived needs meant occupational health service need as felt by migrant workers themselves. “Evaluated need” referred to whether migrants suffered from any diseases in the past 12 months or not. The fourth *Latent variables* are Personal health practices and use of occupational health services, the corresponding *measured variables* of which are weekly physical exercise, use of protective equipments, regular health check-up. The fifth *Latent variables* are Perceived HRQOL, the corresponding *measured variables* of which are PCS and MCS.

### Statistical analysis

The database was constructed using Epidata 3.0. First, to test validity, the factor structure of SF-12 and measured variables of Anderson's behavior's model were extracted by performing exploratory factor analysis (EFA) through the principal component analysis with varimax rotation, respectively. It was hypothesized that a two-factor solution would be obtained with eigenvalues greater than 1 for SF-12 and four-factor solution for measured variables of Anderson behavior's model. The corresponding factor loading equal or greater than 0.4 was considered satisfactory. To test reliability, the internal consistency for summary measures of PCS, MCS and measured variables were estimated using Cronbach's alpha coefficient, and alpha ≥0.60 was considered satisfactory [32]. The study variables' deviations from the normal distribution remain mild with skewness statistics ranging from 0.32 to 1.44 [33]. Therefore, no attempts (e.g., data transformation) were made to improve the distributional properties.

Second, descriptive statistics were calculated for independent variables and statistical significant difference between groups was tested. The *t*-test was used for normally distributed continuous variables, and the chi-squared test was used for binominal variables. Independent variables: demographic characteristics, types of enterprises; Dependent variables: PCS & MCS (*t*-test for two groups or Analysis of variance (ANOVA) for three or more subgroups), migrants' number in different SMEs (chi-squared test). *P*≤0.05 was considered to indicate statistical significance. Statistical analyses were performed using SPSS version 19.0.

Lastly, SEM was employed to test the hypothesized Andersen's behavioral model that specified relationships among predisposing factors, enabling resources, care needs, health practices and use of health services, and health outcome. This analysis involved a two-step process. First, confirmatory factor analysis (CFA) was used to describe the relationships between the latent constructs and their measured variables (measurement model). Standardized factor loadings were calculated for each indicator (the higher, the better). Then, SEM was performed to test the hypothesized relationships among all latent constructs (structural model). The measurement and structural models were examined using AMOS (Analysis of Moment Structures) 18.0. Standardized regression coefficients for all paths were estimated. Goodness of model fit was evaluated by using absolute and comparative fit indices. Missing data accounted for no more than 2% of any variables in this study and were imputed using mean replacement method [34]. A Chi-Square/DF ratio <3.0, a root mean squared error of approximation (RMSEA) <0.06, standardized root mean square residual (SRMR) <0.05, a goodness-of-fit index (GFI), comparative fit index (CFI), and Tacker-Lewis index (TLI) of 0.90 or above were taken to indicate an acceptable model fit [35].

## Results

### Basic characteristics of the study participants

Nearly 40% of the participants in this investigation were female, 80% were at the age of 15 to 39 years old, 36% were undergoing family separation; nearly 41% of China's migrant workers in 2011 were female, 80.4% were at the age of 15 to 39 years old, 35% were undergoing family separation [36]. After the Chi-squared goodness-of-fit test, no significant difference was found between the distributions of sample characteristics (sex/age/family separation) and those of China's migrant workers in 2011 (all *p*-values>0.45).

The social-demographic information of study participants is reported in Table 3. Nearly 60% of the participants were more than 27.9 years old, 80% were educated at or below junior middle school education; 81.3% had changed jobs by the time we conducted the survey, and had resided in more than one place (each over 6 months). Eighty-seven percent migrated from other provinces. There were significantly higher proportion of migrants from other provinces in medium and miniature enterprises than the small enterprises (*P* = 0.014; *P* = 0.015). Over 50% had a monthly income less than RMB3000 (US$473.4). Over 60% of migrants worked more than 8 hours per day. Migrants working in medium enterprises had higher average monthly income (*P*<0.01) and longer working hours per day (*P*<0.01) than those working in the small and miniature enterprises.

**Table 3:** Demographic characteristics and scores of Chinese migrants among different SMEs (*n* = 856).

Variables	Medium *N (%)*	Small *N (%)*	Miniature *N (%)*	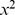	*N (%)*	PCS	*t & F*	MCS	*t & F*
Age									
>27.9	274(60.8)	164(54.5)	67(64.4)	4.37	505(59.0)	53.55±5.07	0.74	52.80±7.08	6.78***
≤27.9	177(39.2)	137(45.5)	37(35.6)		351(41.0)	53.26±5.87		49.17±8.14	
Sex									
Female	208(46.1)	114(37.9)	23(22.1)	21.38**	345(40.3)	53.02±5.78	1.79	49.96±7.83	4.26***
Male	243(53.9)	187(62.1)	81(77.9)		511(59.7)	53.71±5.14		52.23±7.54	
Education									
Primary or less	53(11.8)	29(9.6)	18(17.3)	9.03	100(11.7)	52.77±5.69	1.36	52.57±7.21	2.23
Junior middle school	321(71.2)	201(66.8)	69(66.3)		591(69.0)	53.41±5.37		51.33±7.66	
High school or above	77(17.0)	71(23.6)	17(16.4)		165(19.3)	53.90±5.38		50.50±8.24	
BMI									
Outside the normal range	173(38.4)	135(45.0)	33(31.7)	6.60	341(40.0)	53.02±5.58	−1.79	51.37±7.84	0.14
18.5∼22.9	278(61.6)	165(55.0)	71(68.3)		514(60.0)	53.69±5.29		51.29±7.67	
Household register									
Other Provinces	400(88.7)	248(82.4)	96(92.3)	9.33*	744(87.0)	53.53±5.22	1.22	51.37±7.78	0.53
Guangdong Province	51(11.3)	53(17.6)	8(7.7)		112(13.0)	52.74±6.53		50.96±7.46	
Personal monthly income									
<¥2000	20(4.4)	16(5.3)	23(22.1)	56.83**	59(6.9)	53.81±5.13	1.23	50.19±8.03	7.38
¥2000∼	140(31.1)	67(22.3)	29(27.9)		236(27.6)	52.86±6.00		50.12±7.76	
¥2500∼	84(18.6)	84(27.9)	19(18.3)		187(21.8)	53.53±5.38		50.39±8.17	
¥3000∼	207(45.9)	134(44.5)	33(31.7)		374(43.7)	53.67±5.07		52.71±7.24^△□^	
Hours of work									
>8 h	333(73.8)	174(57.8)	45(43.3)	43.52**	552(64.5)	53.29±5.60	−0.99	50.18±8.08	−5.86***
≤8 h	118(26.2)	127(42.2)	59(56.7)		304(35.5)	53.67±5.06		53.36±6.60	
Ever changed work									
Yes	362(80.3)	242(80.4)	92(88.5)	3.99	696(81.3)	53.43±5.51	−0.07	51.24±7.58	−0.72
No	89(19.7)	59(19.6)	12(11.5)		160(18.7)	53.47±4.93		51.73±8.33	
Number of residences of more than six months									
≤3	9(2.0)	3(1.0)	1(1.0)	8.72	13(1.5)	53.31±5.44	5.91**	51.28±7.82	0.07
4∼6	68(15.1)	54(17.9)	28(26.9)		150(17.5)	54.46±4.53		51.52±7.46	
>6	374(82.9)	244(81.1)	75(72.1)		693(81.0)	49.77±8.93^△▽^		51.00±7.30	
Total	451(52.7)	301(35.2)	104(12.1)		856	53.43±5.42		51.31±7.73	

*P*≤0.05, **P≤0.01, ***P≤0.001, ^△^ *P*≤0.05 (compared with the first option),^▽^ *P*≤0.05 (compared with the second option), ^□^ *P*≤0.01 (compared with the second and third option).

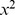
: the statistics for chi-squared test; t: the statistics for t-test; F: the statistics for Analysis of variance (ANOVA); PCS: physical component summary; MCS: mental component summary; h: hour; ¥: the currency symbol of RMB.

Note: Most of the variables' categories follow the national migrants' survey [3]. the mean age of Chinese migrants were 27.9 in 2011,thus the age variable was divided into two categories (>27.9 and ≤27.9). The monthly income of migrants was divided into four group “<¥2000”, “<¥2500”, “<¥3000” and “¥3000+”. Our following results also suggested they were reasonable categorization with an equal sample in each group. Migrants often felt lonely and had less social support when they did not live with their family members. The term “family separation” could convey this meaning, but “divorced or widowed, married, and single” did not. The education was categorized by “primary school or less, junior middle school and high school or above.

Table 4 summarizes the measured indicators of the migrants' characteristics according to Andersen's model. *Predisposing*: for family separation, age and education see above; Enabling: over 40% did not receive occupational health and safety training and physical examination (for income see above); 65.8% had signed the labor contract of no less than one year and 91.7% had bought insurance; *Need*: more than 60% perceived the need of occupational health service; *Health behavior*: more than 60% did not have weekly physical exercise, and regular health check-up, less than 20% used protective equipments during work.

**Table 4:** The latent and measured variables used in the analysis (N = 856).

Variable	*N*/Mean	%/SD	Variable	*N*/Mean	%/SD
***Predisposing factors***			Occupational health and safety training		
Age			No	367	42.9
>27.9	505	59.0	Yes	489	57.1
≤27.9	351	41.0	Physical examination		
Family separation(did not live with their family members)			No	397	46.4
Yes	307	35.9	Yes	459	53.6
No	549	64.1	***Need***		
Education			Perceived occupational health service need		
Primary school or below	100	11.7	Yes	527	61.6
Junior middle school	591	69.0	No	329	38.4
High school or above	165	19.3	Contract a disease in the past 12 month		
***Enabling resources***			Yes	523	61.1
Personal monthly income			No	333	38.9
<¥2000	59	6.9	***Personal health practices and use of occupation health services***		
¥2000∼	236	27.6	Physical exercise weekly		
¥2500∼	187	21.8	No	517	60.4
¥3000∼	374	43.7	Yes	339	39.6
Labor contract			Use of protective equipments		
<1 year	293	34.2	No	690	80.6
1 year∼	312	36.4	Yes	166	19.4
3 years∼	251	29.3	Regular check-up		
Insurance			No	535	62.5
No	71	8.3	Yes	321	37.5
One of the three	351	41.0	***Perceived HRQOL***		
Two of the three	428	50.0	PCS	53.4	5.4
All of the three	6	0.7	MCS	51.3	7.7

SD: standard deviation; ¥: the currency symbol of RMB; PCS: physical component summary; MCS: mental component summary.

### Reliability and validity of SF-12 and measured variables

To test reliability, the SF-12 item and summary descriptive statistics are presented in Table 2. The results demonstrated that both summary measures exceeded the 0.60 level for Cronbach's alpha, indicating satisfactory results (α for the PCS-12 = 0.62; MCS-12 = 0.65). In addition, no floor or ceiling effects were observed, implying that the SF-12 items captured the full range of health status.

To test validity, the two-factor conceptual structure of the SF-12 was confirmed (Table 5). Principal components analysis with varimax rotation loaded two factors showed that eigenvalues for the two factors (physical and mental health) jointly accounted for 60.3% of the variance. The results indicated that items of physical functioning, role physical, bodily pain and general health loaded higher on the physical health component; items of vitality, social functioning, role emotional and mental health loaded higher on the mental health component.

**Table 5:** Factor structure of the SF-12 derived from principal component analysis.

Item description	SF-12 domain	Factor 1	Factor 2
Health in general	General Health	**0.55**	0.45
Moderate activities	Physical Functioning	**0.69**	0.10
Climb several flights	Physical Functioning	**0.72**	0.10
Accomplished less	Role Physical	**0.60**	0.23
Limited kind of work	Role Physical	**0.56**	0.29
Pain interferes	Bodily Pain	**0.65**	0.35
Accomplished less	Role Emotional	0.25	**0.77**
Not careful	Role Emotional	0.22	**0.77**
Peaceful	Mental Health	0.25	**0.65**
Blue/sad	Mental Health	0.28	**0.64**
Energy	Vitality	0.27	**0.55**
Social time	Social Functioning	0.18	**0.68**
Variance explained (%)		37.49	22.80

Measured variables of Anderson's behavior's model had good reliability with most of domains ≥0.60 levels for Cronbach's alpha; and good validity with the item loading higher on the corresponding factor than on other factors (Table 6).

**Table 6:** The reliability and validity of measured variables (N = 856).

Item description	Domain	Cronbach's α	Factor 1	Factor 2	Factor 3	Factor 4
Age	Predisposing	0.60	0.64	0.24	0.10	−0.22
Family separation	Predisposing		0.67	0.06	−0.06	0.06
Education	Predisposing		−0.58	0.19	−0.04	0.23
Personal monthly income	Enabling	0.57	−0.01	0.67	−0.06	−0.12
Labor contract	Enabling		−0.03	0.71	−0.21	−0.12
Type of insurance purchase	Enabling		−0.03	0.63	0.14	0.03
Physical examination during the work	Enabling		0.34	0.56	0.08	0.15
Occupational health and safety training	Enabling		0.16	0.40	0.25	0.31
Perceived occupational health service need	Need	0.65	−0.09	−0.31	0.71	0.11
Contract a disease in the past 12 month	Need		0.07	−0.11	0.63	−0.23
Physical exercise weekly	health practices and use of health services	0.63	−0.25	−0.01	0.07	0.69
Use of protective equipments	health practices and use of health services		0.08	−0.05	−0.02	0.68
Regular check-up	health practices and use of health services		−0.20	−0.02	−0.09	0.77

### HRQOL scores

The study participants had an average score of 53.4 for PCS and 51.3 for MCS, respectively. migrants who were male, over 27.9, had statutory working time (≤8 hours/day) or a monthly income of over 3000 Yuan had a higher MCS score, than the migrants who were female, less than 27.9, did not have statutory working time or had a monthly income lower than 3000 Yuan. Migrants working in medium enterprises or having resided in more than 6 places(each over 6 months) had a lower PCS than those working in small-and miniature enterprises or having less than 6 residence locations(Table 3).

### Confirmatory factor analysis

**Figure 2:**
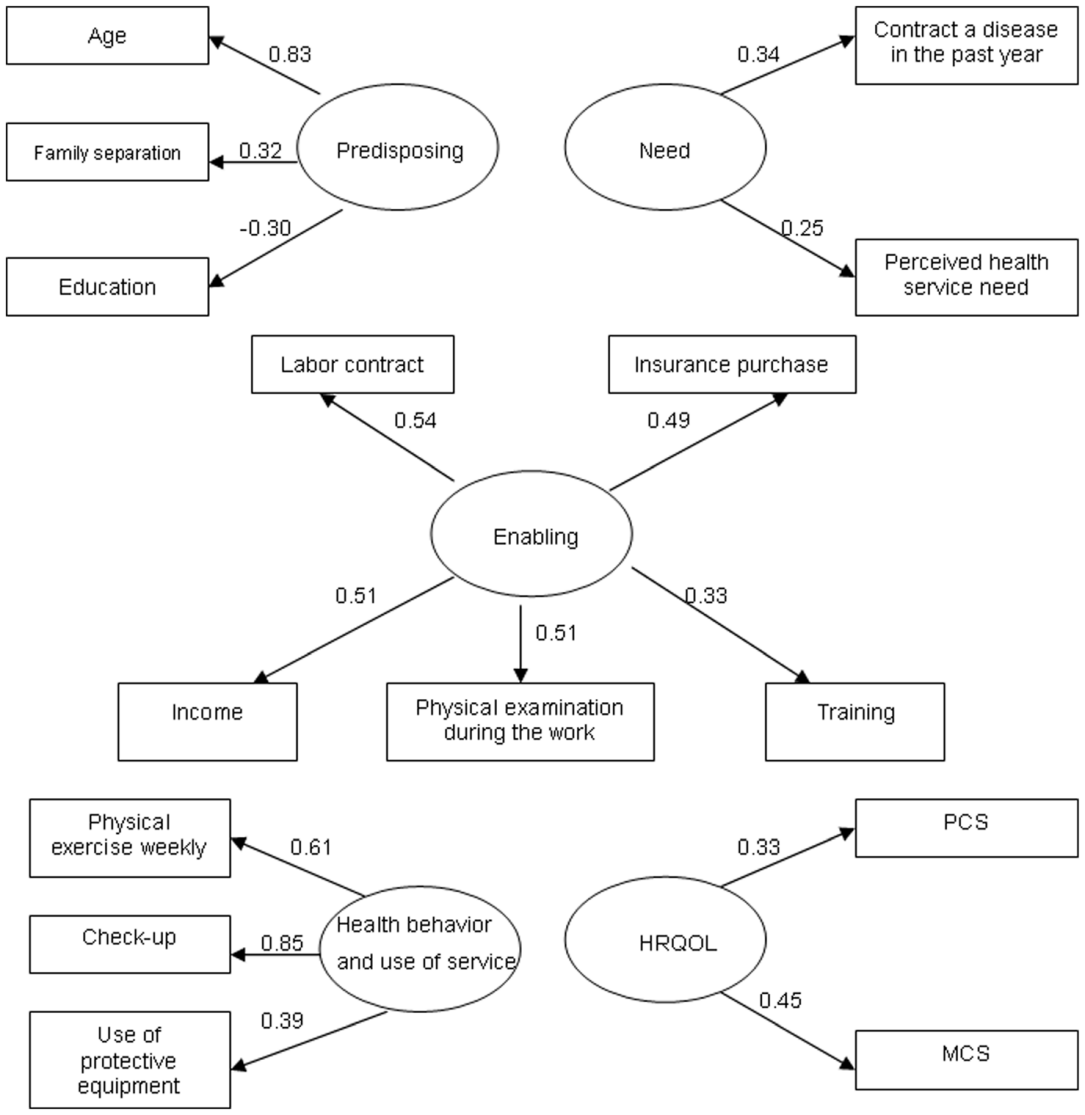
Measurement model of latent constructs (ellipses) and manifest indicator variables (rectangles). Values represent standardized factor loadings and all are statistically significant (*P*<0.01). GFI = 0.973; CFI = 0.925; TLI = 0.886; RMSEA = 0.045; SRMR = 0.045. Chi-Square/DF = 2.736.

The revised measurement model indicated a GFI of 0.973, CFI of 0.925, TLI of 0.886, RMSEA of 0.045, SRMR of 0.045 and Chi-Square/DF of 2.736,suggesting an acceptable model fit (Fig. 2).

The predisposing factor was highly associated with age, family separation and education. Of these, age had the highest factor loading (0.83). The enabling factor was highly associated with the status of contract, insurance, physical examination and income. The need factor was highly associated with being ill in the past year and perception of needing health service. The health behavior and use of services factor was highly associated with frequency of physical exercise, regular check-up and using protective equipments. The perceived health outcome factor was highly associated with PCS and MCS (Fig. 2). The correlations between the five latent factors ranged between −0.497 and 0.781, indicating that they had acceptable discriminant validity (i.e. <0.85) [37].

### Structural model analysis

**Figure 3:**
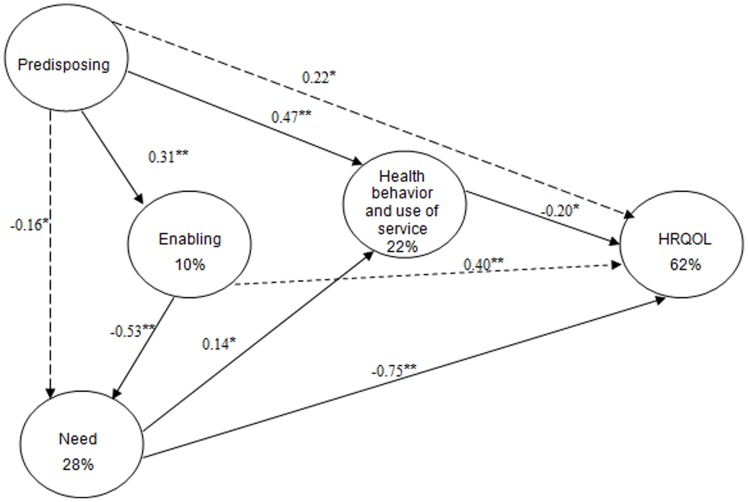
Structural model for Andersen's behavioral model. Standardized path coefficients are shown. *P <0.05; **P <0.01. GFI = 0.967; CFI = 0.886; TLI = 0.855; SRMR = 0.047; RMSEA = 0.047. Chi-Square/DF = 2.916. Solid lines = direct effect; dashed lines = indirect effect.

The initial hypothesized model presented four non-significant paths: predisposing→ need, predisposing→ health outcome, enabling→ behavior, and enabling→ health outcome. We first eliminated these four non-significant paths. Further tests suggested that removing these non-significant paths would decrease the chi-square of the model and thus increase the model fit. The revised model was then estimated and the overall fit of the revised model was good: GFI = 0.967; CFI = 0.886; TLI = 0.855; SRMR = 0.047; RMSEA = 0.047. Chi-Square/DF = 2.916 (Fig. 3).

In this revised model, twenty-eight percent of the variance of the need factor was explained by the predisposing and enabling factors. Ten percent of the variance of the enabling factor was explained by the predisposing factor. Twenty-two percent of the variance of health behaviour and use of services factor was explained by the predisposing and need factors. Sixty-two percent of the HRQL was explained by need, predisposing, heath behavior and enabling factors.

### Direct, indirect and total effects

*Direct, indirect and total effects of the latent variables were illustrated in* Table 7 and Figure 3. Most of the direct effects were under expectation. Greater predisposing factor was associated with more enabling resources (*β* = 0.31); greater enabling resources were associated with less need (*β* = −0.53); Greater need was associated with positive behavior and greater use of services (*β* = 0.14) and less HRQOL (*β* = −0.78). Surprisingly, worse behavior and less use of services were associated with better perceived HRQOL.

**Table 7:** Direct, indirect and total effects for Andersen's model.

	*Predisposing*	*Enabling*	*Need*	*Health behavior and use of service*
***Enabling***				
Direct effect	0.31**			
Indirect effect				
Total effect	0.31**			
***Need***				
Direct effect		−0.53**		
Indirect effect	−0.16*			
Total effect	−0.16*	−0.53**		
***Health behavior and use of service***				
Direct effect	0.47**		0.14*	
Indirect effect	−0.02	0.08		
Total effect	0.45**	0.08	0.14*	
***HRQOL***				
Direct effect			−0.75**	−0.20*
Indirect effect	0.22*	0.40**	−0.03	
Total effect	0.22*	0.40**	−0.78**	−0.20*

*P*<0.05; **P<0.01.

*Direct negative effect*: health behavior and use of service on HRQOL (*β* = −0.20); need on HRQOL (*β* = −0.75); enabling on need (*β* = −0.53).

*Indirect effect*: predisposing factor on HRQOL mediated by enabling, need and health behavior and use of service; enabling factor on HRQOL mediated by need and health behavior and use of service.

*Total effect*: Need had greater total effect on HRQOL (*β* = −0.78), followed by enabling (*β* = 0.40), predisposing (*β* = 0.22) and health behavior and use of service (*β* = −0.20).

## Discussion

Our results suggest that Andersen's behavioral model is a useful model in predicting the HRQOL among rural-to-urban migrants in SMEs in China. In our study, variables related to workers' health, such as need for occupational health service, use of protective equipments, and occupational health training, were included in the model. Ten percent, 28%, 22% and 62% of the variance of the enabling resources, need, personal health practices and use of services, and perceived HRQOL could be explained by the related latent variables respectively. In consistent with another study, our study suggests good predicating power of the adapted Andersen model [38]. In particular, this model has the best predictability for perceived HRQOL, more than 50% of the variance of which could be explained by other factors in this model. Our study indicates the importance of sufficient enabling resources and small need in predicting good HRQOL. Our results echo other studies[38]–[41], which adapted the Andersen's model to examine the direct and mediated pathways between social, attitudinal and behavioral factors and perceived oral health outcomes. However, those studies have confined themselves in traditional methods, not the SEM. Use of SEM can explore the correlation and recursive relationships between these key factors and the HRQOL outcome.

Our results highlight the importance of testing complex interrelationships between key contextual factors when examining migrant HRQOL. In this model, predisposing factor has direct effect on enabling factors, indicating the positive associations between the predisposing factors such as age, education and enabling variables such as income, insurance, labor contract. Enabling factors have a negative direct effect on need factors, while predisposing factors have negative indirect effect on need factors mediated by enabling factors. In line with Andersen's model, if one is more predisposed to seek care, they need to have access to the enabling resources [40], [42].

In our study, health behavior factor has a negative direct effect on HRQOL. The health behavior factor was found to be more associated with health check-up (factor loading 0.85) than others (Fig. 2). This suggests that health check-up dominated the direction of relationship between health behavior and health outcome. In reality, weekly physical exercise and use of protective equipment have closer relations (closer in time to the health outcome) with HRQOL than health check-up once or twice a year (more distant in time). Thus, check-up leads to weak or negative effect in the causal chain between health behavior and perceived HRQOL.

Echoing with previous published studies [43], [44], our study suggests that migrants who had statutory working time (≤8 hours/day) or higher income had a higher MCS score than migrants who did not have statutory working time or had lower income; Migrants who had less migrant experience had higher PCS score than migrants who had more migrant experience. In our complementary analysis, migrants in the medium enterprise had higher income, worked in longer hours per day and had a less PCS than those working in the small and miniature enterprise. Less PCS among migrants in medium enterprises might be due to larger business volume, more heavy workload and more stringent management. Therefore, providing enough labor protection and social security for workers will benefit the health of migrants especially those working in medium enterprises. Despite use of protective equipment helps to protect physical health, less than 20% migrant workers used it in this study. Wearing protective equipments might slow down the working efficiency of migrants, who seek better livelihood in the city. Also, SMEs may seek profit at the sacrifice of workers' health by not investing. Limited use of protective equipments may contribute to the poor HRQOL scores of migrants.

Policy implications can be drawn from our study to improve the HRQOL of migrant workers. First, SMEs should satisfy the need of occupational health service (OHS) of migrants. Education program should be provided to improve the social responsibility of the employers [45], [46]. Essential OHS services such as protective equipments should be provided to protect migrants' health. Second, governments and employers should ensure the enabling resources to be provided to migrant workers. It is imperative to provide migrants with security benefits such as labor contracts and insurances. Regular OHS training and physical examinations will improve the self-protection awareness of migrant workers and early detection of health problems [47]. Things will be much improved, following the implementation of universal basic occupational health service (BOHS) [48].

Our study is one of the first to apply Andersen's behavior model and SEM to analyze potential determinants of HRQOL of migrant workers in SMEs. Several limitations should be born in mind. First, our study is cross-sectional; such that causality cannot be determined. Using this model entails more creative and challenging conceptualization, longitudinal and experimental study designs, and innovative types of statistical analyses [19]. Some important factors may have been neglected in the model, such as personal beliefs or biological risk factors [49]. Second, Cronbach's alpha of 0.60 in this study is not very strong. As most of the migrants were young, they may not have some problems reflected in SF-12 (e. g. Pain interferes (BP) or Climb several flights (PF2), etc.). The inconsistency of choosing the answers of SF-12 between the old and the young migrants leads to its low reliability. Finally, future research is needed to identify more variables to explain the causal relationships among the variables of Anderson's behavior's model.
